# Genetic variants (*Lhcgr*^W495X/+^) and environmental toxicants (DEHP) synergistically induce DSD by interfering with steroidogenic gene expression

**DOI:** 10.1186/s13293-025-00753-0

**Published:** 2025-09-26

**Authors:** Xie Qigen, Xia Kai, Cao Haiming, Xu Zhe, Gao Yong, Deng Chunhua

**Affiliations:** 1 Department of Pediatric Surgery, First Affiliated Hospital, Sun Yat-Sen University, 58 Zhongshan Road 2, Guangzhou, 510080 China; 2 Department of Andrology, First Affiliated Hospital, Sun Yat-Sen University, 58 Zhongshan Road 2, Guangzhou, 510080 China; 3 Department of Andrology, Reproductive Center of the Seventh Affiliated Hospital, Sun Yat-Sen University, Shenzhen, 518000 China; 4 Reproductive Medicine Center, The Key Laboratory for Reproductive Medicine of Guangdong Province, The First Affiliated Hospital of Sun Yat-sen University, Guangzhou, 510080 Guangdong China

**Keywords:** Disorders/differences of sex development, Synergistic effect, DEHP, *LHCGR*

## Abstract

**Background:**

Emerging evidence suggests that genetic variants and environmental toxicants may synergistically contribute to DSD. To test this hypothesis, we employed *Lhcgr*^W495X/+^ (luteinizing hormone/chorionic gonadotropin receptor) male mice subjected to prenatal Di-(2-ethylhexyl) phthalate (DEHP) exposure, a model designed to investigate steroidogenic gene expression in gene-environment interactions.

**Methods:**

Pregnant wild-type (WT) dams (mated with *Lhcgr*^W495X/+^ heterozygote (HET) received varying levels of DEHP: no exposure, low-dose (100 mg/kg/d) DEHP, and high-dose (1000 mg/kg/d) DEHP during gestation, which led to prenatal exposure in male offspring. Male offspring were divided into HET (*Lhcgr*^W495X/+^) and WT groups based on genotype in three levels of DEHP exposure. The study assessed phenotypic characteristics (DSD, testosterone levels, and semen quality) and examined the expression of steroidogenic genes (*Lhcgr*,* Star*,* Cyp11a1*,* Cyp17a1*,* Hsd17b3*, and *Hsd3b2*).

**Results:**

*Lhcgr*^W495X/+^ male offspring without DEHP exposure exhibited normal phenotypes and steroidogenic gene profiles. Low-dose DEHP had no detectable effects on WT offspring, but synergistically induced DSD in *Lhcgr*^W495X/+^ male offspring by interfering with steroidogenic gene expression (*Lhcgr*,* Hsd17b3*,* Hsd3b2*). High-dose DEHP caused DSD in both genotypes, but the severity of DSD and interference with steroidogenic gene expression were more pronounced in *Lhcgr*^W495X/+^ male offspring.

**Conclusions:**

This study verifies that Genetic variants (*Lhcgr*^W495X/+^) and environmental toxicants (DEHP) synergistically induce DSD, thereby elucidating the pathogenesis of DSD. Interfering with steroidogenic gene expression may be an important synergistical mechanism. This finding highlights the clinical imperative to minimize prenatal exposure to endocrine disruptors, particularly in pregnancies with variants of DSD.

**Supplementary Information:**

The online version contains supplementary material available at 10.1186/s13293-025-00753-0.

## Highlights

- This study originally constructs a DSD model involving both genetic and environmental factors, *Lhcgr*^W495X/+^ male mice with prenatal DEHP exposure. This model sufficiently substantiates the theory that genetic variants and environmental toxicants synergistically induce DSD.
- Prenatal exposure to high-dose DEHP exacerbated DSD in *Lhcgr*^W495X^ offspring, demonstrating a dose-dependent synergistic effect between genetic variants and environmental exposure.
- Our investigation reveals the pivotal role of nonpathogenic variants in DSD. Genetic susceptibility amplifies environmental toxicity. Fetuses with nonpathogenic variants of DSD are more sensitive to EEDs.
- Clinically, our findings highlight the clinical imperative to minimize prenatal exposure to endocrine disruptors, particularly in pregnancies with variants of DSD.

**Supplementary Information:**

The online version contains supplementary material available at 10.1186/s13293-025-00753-0.

## Plain language summary

The pathogenesis of disorders/differences of sex development (DSD) remains incompletely understood. While genetic variants and environmental toxicants are hypothesized to synergistically contribute to DSD, no animal model has been established to validate this interaction, and the mechanistic basis remains elusive. Here, we propose that such synergy may impair steroidogenic gene networks, thereby disrupting testosterone synthesis and inducing DSD. To test this, we employed *Lhcgr*^W495X/+^ (luteinizing hormone/chorionic gonadotropin receptor) male mice exposed prenatally to di-(2-ethylhexyl) phthalate (DEHP), a model recapitulating human gene-environment interplay. Pregnant dams (mated with *Lhcgr*^W495X/+^ males) received no, low-dose(100 mg/kg/d), or high-dose(1000 mg/kg/d) DEHP during gestation, which led to prenatal exposure in male offspring. Male offspring were divided into HET (*Lhcgr*^W495X/+^) and WT groups by genotype (*Lhcgr*^W495X/+^ or WT) in three levels of DEHP exposure. This design enabled simultaneous evaluation of genetic (*Lhcgr*) and environmental (DEHP) effects on male offspring. Notably, male offspring carrying the *Lhcgr*^W495X/+^ variant or exposed to low-dose DEHP alone exhibited normal phenotypes. However, prenatal exposure to low-dose DEHP selectively induced DSD in *Lhcgr*^W495X/+^ male offspring via interfering with steroidogenic gene expression (*Lhcgr*,* Hsd17b3*,* Hsd3b2*). High-dose DEHP caused DSD in both genotypes, but with significantly greater severity in *Lhcgr*^W495X/+^ offspring. In conclusion, our findings suggest that genetic variants (*Lhcgr*^W495X/+^) and environmental toxicants (DEHP) synergistically disrupt steroidogenesis, leading to DSD. Clinically, our findings underscore the need for rigorous avoidance of prenatal endocrine disruptors, particularly in pregnancies with variants of DSD.

**Supplementary Information:**

The online version contains supplementary material available at 10.1186/s13293-025-00753-0.

## Background

Disorders/differences of sex development (DSD) encompass a broad range of congenital genital malformations in which the development of chromosomal, gonadal, or anatomical sex is atypical [1]. The most prevalent type among these disorders is 46,XY DSD, presenting phenotypes such as hypospadias, cryptorchidism, micropenis, and ambiguous external genitalia [2]. The irreversibility of DSD in adulthood underscores the critical need for early preventive strategies. Emerging evidence implicates genetic variants and prenatal exposure to environmental endocrine disruptors (EEDs) as primary etiological factors [3–5]. However, definitive pathogenic variants or specific toxicants are identified in less than 30% of cases, leaving the majority of DSD pathogenesis unexplained [6, 7]. Fetuses within the DSD spectrum are often affected simultaneously by genetic and environmental factors in utero. Previous investigations have discovered the synergistic effects of genetic and environmental factors in the pathogenesis of disorders like hypospadias and autism [4, 8, 9]. Therefore, this study posits the hypothesis that DSD results from the synergistic interplay of genetic and environmental factors, although pertinent research and models have hitherto been lacking.

Steroidogenic gene variants are pivotal in 46,XY DSD etiology [10]. The luteinizing hormone/choriogonadotropin receptor (*LHCGR*) orchestrates testosterone synthesis by activating downstream steroidogenic effectors: *STAR* (cholesterol transport), *CYP11A1* (cholesterol side-chain cleavage), *CYP17A1* (17α-hydroxylase/17,20-lyase), *HSD17B3* (testosterone synthesis), and *HSD3B2* (Δ⁵-Δ⁴ isomerase) [11–14]. DSDs could be found in patents with the homozygous and compound heterozygous mutants of *LHCGR* after birth [10, 15]. However, heterozygous variants of *LHCGR* are typically considered nonpathogenic, they represent a genetic susceptibility factor for 46,XY DSD, Our former study has shown that *Lhcgr*^W495X^ knock-in point mutation (harboring a human-relevant point mutation) caused DSD in male mice, however, *Lhcgr*^W495X/+^ mice manifested as normal sex development in adult stage [16]. Therefore, to model the genetic susceptibility, we established *Lhcgr*^W495X/+^ mice to simulate clinical patients. Di-(2-ethylhexyl) phthalate (DEHP), a ubiquitous plasticizer in medical devices and consumer products [17], is a potent anti-androgenic EED [18]. Prenatal DEHP exposure suppresses fetal testosterone synthesis via inhibition of steroidogenic genes expression, culminating in hypospadias and cryptorchidism in rodents [19, 20]. This makes DEHP an ideal agent to probe gene-environment synergies in 46,XY DSD. Thus, we constructed a DSD model employing *Lhcgr*^W495X/+^ mice with prenatal DEHP exposure to substantiate the postulate that gene-environment synergies underlie the pathogenesis of DSD.

Testosterone synthesis involves a series of steroidogenic genes [11–14]. Environmental factors induce Leydig Cell Death and Senescence and testis toxicity, thus interfere with steroidogenic gene expression [21–23]. Most genetic factors of DSDs also interfere with steroidogenic gene expression [10]. Hence, interference with steroidogenic gene expression may constitute a pivotal synergistic mechanism. Previous studies showed that prenatal high-dose DEHP exposure can disrupt steroidogenic gene expression [24–27]. In parallel, the presence of *Lhcgr* heterozygous variants diminishes the efficacy of steroidogenic gene expression. Therefore, prenatal DEHP exposure may synergistically induce DSD by interfering with the expression of steroidogenic genes in *Lhcgr*^W495X/+^ male mice. In addition, exploring the dosage-dependent impact on phenotypic outcomes and steroidogenic gene expression within *Lhcgr*^W495X/+^ male mice, thus augmenting our understanding of the synergistic effects. Therefore, this study aims to validate the hypothesis that genetic variants and environmental toxicants synergistically induce DSD by employing *Lhcgr*^W495X/+^ male mice exposed to prenatal DEHP, explore the role of interference with steroidogenic gene expression within the context of these synergistic effects, and examine the dose-dependent effects of exposure on this synergistic effect.

## Methods

### Animal treatment, measurement, and sample harvesting

#### Animal treatment

C57BL/6JGpt female (wild type (WT), eight weeks old) and male (*Lhcgr*^W495X/+^, heterozygote (HET), eight weeks old) mice were purchased from Biotechnology corporation (Jicui yaokang, Jiangsu, China). The methodology of the *Lhcgr*^W495X/+^ mouse line was identical to our previous study [16]. Mice were housed in the Specific Pathogen Free animal center at Sun Yat-sen University under a 12-hour light/dark cycle. Female (WT) and male (HET) mice were mated (1:2) in separate cages. A vaginal sperm plug confirmed successful mating. The pregnant mice were subsequently housed separately and treated by gavage daily with the vehicle (corn oil) alone or vehicle containing 100 (low-dose) and 1000 (high-dose) mg/kg/d DEHP (Sigma-Aldrich, 36735-1G, USA) from Gestational Day 10 to Postnatal Day 0 (PND0), which covers a critical period for genital development and differentiation in male mice [28]. Doses were adjusted daily according to body weight. The male offspring were divided into WT and HET (*Lhcgr*^W495X/+^) groups according to the genotype, with the two groups thereby serving as littermate controls. The male offspring were anesthetized using pentobarbital sodium and then sacrificed by cervical dislocation, and observed in the neonatal (PND1, mainly for exploring mechanism) and adult (PND56, mainly for observing the phenotype) stages (Supplementary Fig S1).

#### Measurement and sample harvesting

All sacrifices and measurements were performed in the afternoon. The mice and its testes in each litter were assigned to WT and HET groups according to genotype, thus the mice in these two groups could be compared in pairs in some degree.

At PND1, twenty male offspring were sacrificed in each group. The birth weight, testis weight, anogenital distance (AGD), and penile length were measured (*n* = 20). The bilateral testes were harvested. The testes of 5 mice were selected randomly for intratesticular testosterone detection. The testes of the remaining 15 mice were for processed for Reverse Transcription Qualitive Polymerase Chain Reaction (RT-qPCR) and Western Blotting (WB) analysis; Due to the testis was too small, three testes (one from each of three individual mice) on the same side were pooled as one sample, thus we acquired 5 right testis samples for qPCR and 5 left testis samples for WB analysis.

The remaining mice (10–15 in each group) were fed till PND56. Then the mice were sacrificed and dissected. The body weight, AGD, weights of genital organs (testis, epididymis, and seminal vesicle), and penile length were measured (*n* = 10). AGD was measured as the distance (in mm) from the center of the anus to the base of the penis using a digital caliper (Deli, Hangzhou, China; precision: 0.01 mm). Blood samples were collected from the inferior vena cava and prepared for testosterone detection (*n* = 10). The right testis was prepared for qPCR (*n* = 10), and the left was prepared for WB (*n* = 5) and hematoxylin–eosin (HE) staining (*n* = 5).

### DNA extraction and identification of Lhcgr genotype

Total DNA was extracted from mice tails using a DNA extraction kit (Invitrogen DNAzol Reagent, Thermo Fisher, USA) according to the manufacturer’s instructions. The extracted DNA was amplified and sent to the sequencing company (KingMed Diagnostics Group Co., Ltd. Guangzhou, China) for the identification of the *Lhcgr* genotype. The genotype was determined according to the base peak of the mutation site (as shown in Supplementary Fig S2).

## RNA isolation and RT-qPCR analysis

NucleoZOL (MN, 740404.200, USA) was used to extract the total RNA of the testis following the manufacturer’s instructions. RNA concentration was measured and adjusted using the Nanodrop ONE System (Thermo Scientific, USA). Complementary DNA was then synthesized from 500ng RNA using a PrimeScript™ RT Master Mix (Takara, RR036A, Beijing, China). Subsequent RT-qPCR analysis of steroidogenic genes (*Lhcgr*,* Star*,* Cyp11a1*,* Cyp17a1*,* Hsd17b3*, and *Hsd3b2*) was performed using a Hieff^®^ qPCR SYBR^®^ Green Master Mix (High Rox) (Yeasen,11203ES08, Shanghai, China) on the StepOnePlus RT PCR System (Applied Biosystems, USA). The relative expression of target genes was calculated using the 2^−ΔΔCt^ algorithm. We used *Gapdh* as the reference gene for all target genes. The primers for the genes are listed in Table 1.

**Table 1:** Primer sequences

Primer name	Primer sequences	Length
Lhcgr-F	AATGAGTCCATCACGCTGAAAC	22
Lhcgr-R	CCTGCAATTTGGTGGAAGAGA	21
Star-F	ATGTTCCTCGCTACGTTCAAG	21
Star-R	CCCAGTGCTCTCCAGTTGAG	20
Cyp17a1-F	GCCCAAGTCAAAGACACCTAAT	22
Cyp17a1-R	GTACCCAGGCGAAGAGAATAGA	22
Hsd17b3-F	ATGGGCAGTGATTACCGGAG	20
Hsd17b3-R	ACAACATTGAGTCCATGTCTGG	22
Cyp11a1-F	AGGTCCTTCAATGAGATCCCTT	22
Cyp11a1-R	TCCCTGTAAATGGGGCCATAC	21
Hsd3b2-F	GGTTTTTGGGGCAGAGGATCA	21
Hsd3b2-R	GGTACTGGGTGTCAAGAATGTCT	23
Gapdh-F	AGGTCGGTGTGAACGGATTTG	21
Gapdh-R	TGTAGACCATGTAGTTGAGGTCA	23

## Protein extraction and WB analysis

The method is similar to our previous study [24]. Protein extraction of the testes was performed using RIPA lysis buffer and a 10% protease inhibitor cocktail (MCE, HY-K0010, USA). Lysates were centrifuged for 30 min at 12,000 × *g* at 4 ◦C, and supernatants were collected. Protein concentration was measured using the Pierce™ BCA Protein Assay Kit (Thermo Scientific, 23227, USA) on a microplate reader (562 nm, BioTech Epoch, USA). The protein sample was adjusted to a concentration of 30 µg/20 µl with water and 5X loading buffer (Beyotime, P0015, Shanghai, China). Each track was loaded with 30 µg/20 µl proteins. The proteins were separated by SDS-PAGE gel electrophoresis and transferred to PVDF membranes (0.45 μm, Merck Millipore, IPVH00010, Germany). The membranes were sealed with 5% milk and then washed five times using Tris-buffered saline with Tween 20 (TBST). For western blot analysis, the membranes were incubated with primary antibodies against LHCGR (SAB, 44352, USA), HSD3B2 (Affinity, DF6639, USA), HSD17B3 (SAB, 31304, USA), or β-Tubulin (SAB, 48659, USA) at 4 ℃ overnight. The primary antibody was diluted in a 1:1000 ratio. The membranes were washed five times with TBST. They were then incubated with corresponding secondary antibodies at 25℃ for 1 h. The secondary antibody was diluted in a 1:5000 ratio. Finally, an Immobilon Western Kit (Merck Millipore, WBKLS0500, Germany) was used to display the protein band. β-Tubulin was used as the reference protein.

## Testosterone detection

The blood sample was centrifuged for 10 min at 5000 × *g* at 4 ◦C to separate the serum. The bilateral testes (approximately 4 mg) of neonatal mice were lysed in 100 µl RIPA lysis buffer for 30 min. Then they underwent centrifugation for 30 min at 12,000 × g at 4 ◦C, after which the supernatants were collected. The serum and supernatants were sent to a company (KingMed Diagnostics Group Co., Ltd. Guangzhou, China) for testosterone detection. Chemiluminescence Microparticle Immuno Assay (CMIA) was applied using ARCHITECT 2nd Generation Testosterone Reagent Kit (Abbott, 2P13.23, USA) following the manufacturer’s instructions. The coefficient of variation (CV) of CMIA is 2–5.1% for intra-assay precision and 2.6–5.2% for inter-assay precision. The minimum detectable dose of testosterone is 0.01ng/ml.

## HE staining of testis

The resected testes were fixed overnight in Bouin’s solution (Biosharp, P0111, Hefei, China), dehydrated in 75% ethanol, embedded in paraffin, and sectioned at 5 μm. The sections were deparaffinized with xylene, hydrated with graded ethanol, and stained with HE for histological analysis using an inverted-phase contrast microscope (Olympus, IX83, Japan).

### Semen analysis

After dissecting the mice, the caudal epididymis was shredded and incubated in the sperm incubation solution at 37 °C for 30 min. Then, the sperm suspension was placed in the counting chamber. Sperm count, concentration, and motility were measured using a computer-assisted sperm analysis system (SPERM CLASS ANALYZER).

### Statistical analysis

Results are presented as the mean and standard error of the mean (SEM). The two groups were compared using an unpaired *t*-test or chi-square test. GraphPad Prism 8.0 (San Diego, USA) software was used for all statistical analyses. *p <* 0.05 was considered statistically significant.

## Results

### Lhcgr^W495X/+^ male offspring showed normal phenotype and steroidogenic gene expression

#### Lhcgr^W495X/+^ male offspring showed normal phenotype and steroidogenic gene expression in the neonatal stage

**Fig. 1:**
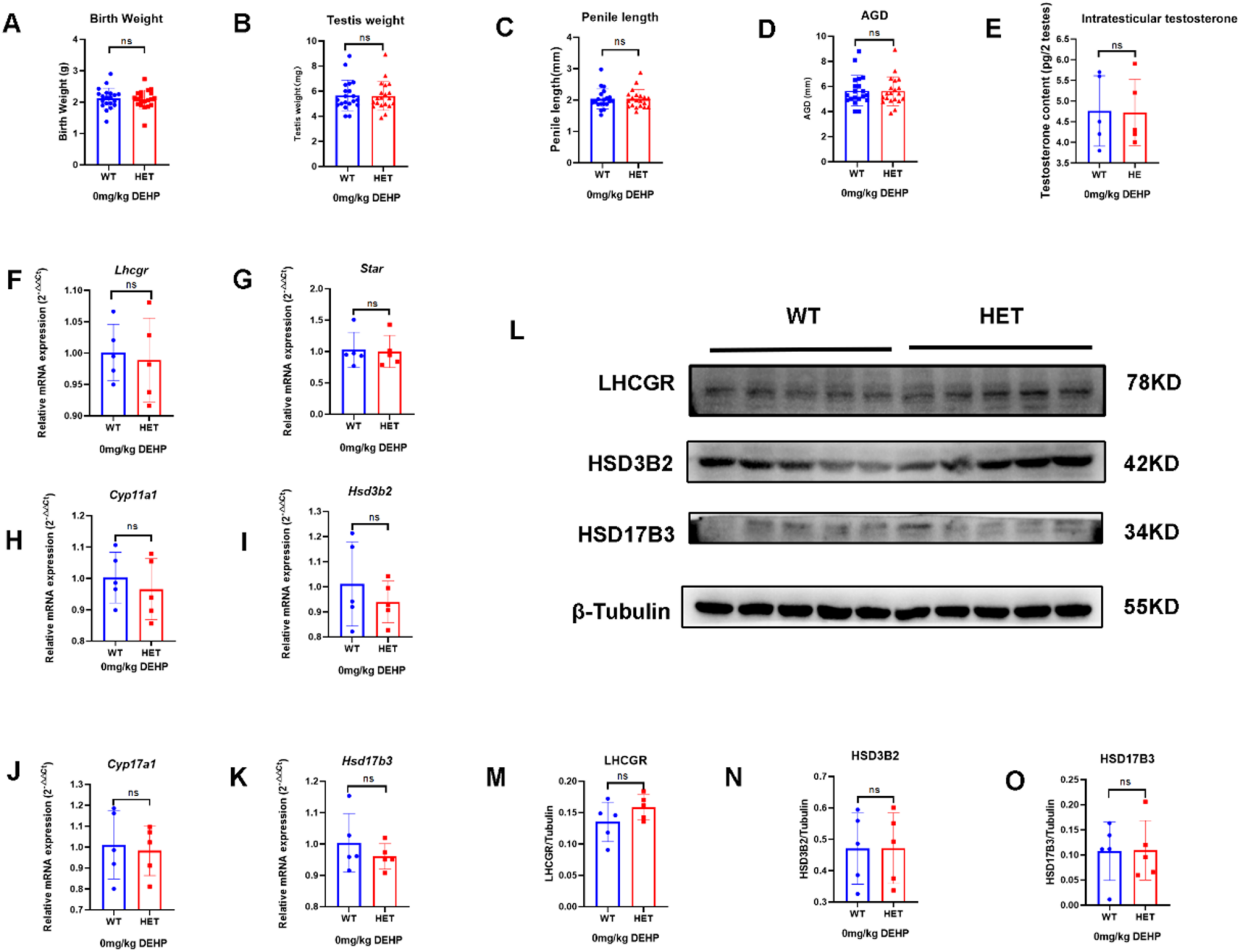
*Lhcgr*^W495X/+^ male mice showed normal phenotype and steroidogenic gene expression in the neonatal stage. **A**-**E**: there was no significant difference in birth weight, testis index, penile length, AGI, and intratesticular testosterone between WT and HET groups. **F**-**K**: there was no significant difference in mRNA expression of steroidogenic genes (*Lhcgr*, *Star*, *Cyp11a1*, *Hsd3b2*, *Cyp17a1*, and *Hsd17b3*) between WT and HET groups. **L**-**O**: there was no significant difference in LHCGR, HSD3B2, and HSD17B3 expressions between WT and HET groups. ns: no significance

In the neonatal stage, no DSD (hypospadias or micropenis) was found in the WT and HET groups. The two groups had no significant differences in birth weight, testis weight, penis length, AGI, and intratesticular testosterone (Fig. 1A-E). There was no significant difference in mRNA expression of steroidogenic genes (*Lhcgr*, *Star*, *Cyp11a1*, *Hsd3b2*, *Cyp17a1*, and *Hsd17b3*) in the neonatal mice between the two groups (*n* = 5, *p* > 0.05) (Fig. 1F-K). WB analysis also confirmed no significant differences between the two groups in the expression of LHCGR, HSD3B2, and HSD17B3 (Fig. 1L-O). Therefore, *Lhcgr*^W495X/+^ male offspring showed normal phenotype and steroidogenic gene expression in the neonatal stage.

#### Lhcgr^W495X/+^ male offspring showed normal phenotype and steroidogenic gene expression in the adult stage

**Fig. 2:**
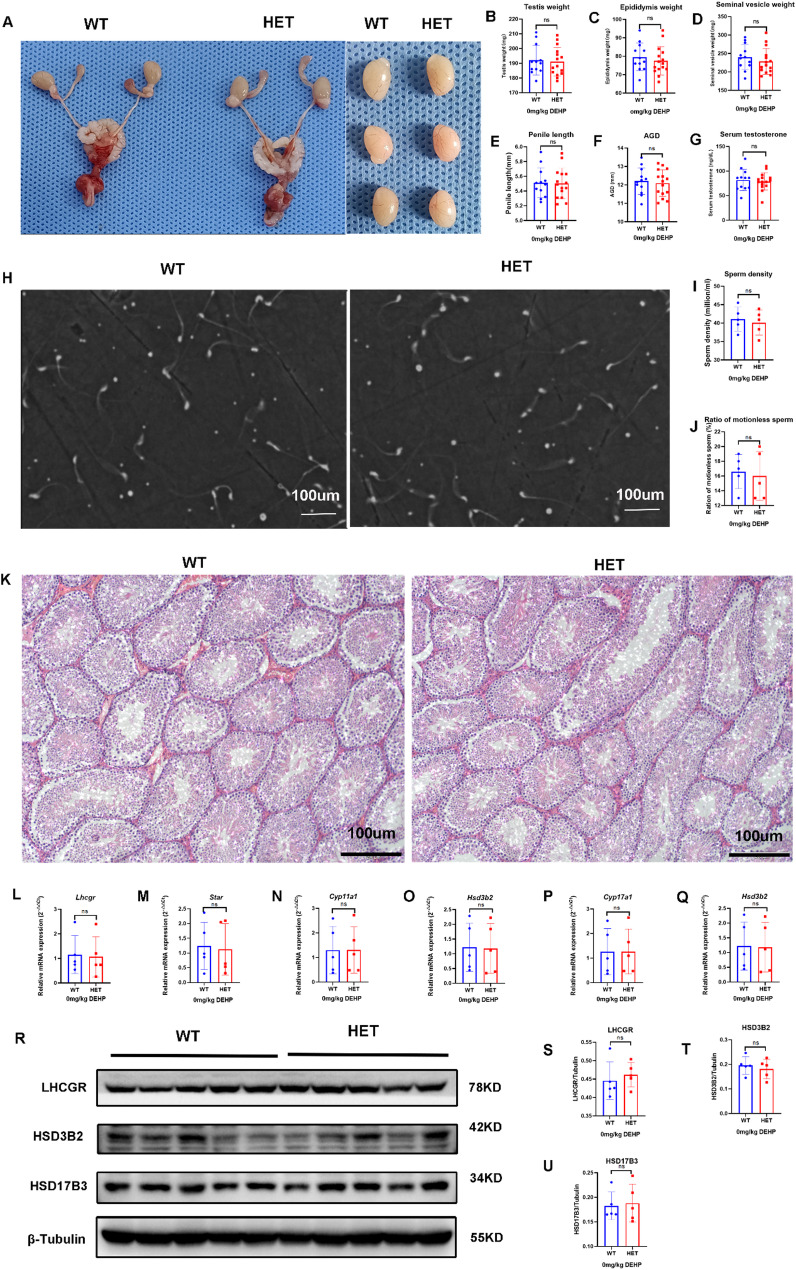
*Lhcgr*^W495X/+^ male mice showed normal phenotype and steroidogenic gene expression in the adult stage. **A**-**G**: there was no difference in genital development and serum testosterone between the two groups. **H**-**J**: there was no significant difference in semen quality between the two groups (X100). **K**: HE staining (X100) of testicular tissue showed no significant difference in the development of seminiferous tubules between the two groups. **L**-**Q**: the two groups showed no difference in mRNA expression of steroidogenic genes (*Lhcgr*, *Star*, *Cyp11a1*, *Hsd3b2*, *Cyp17a1*, and *Hsd17b3*). R-U: the two groups showed no difference in expressions of LHCGR, HSD3B2, and HSD17B3. ns: no significance

In the adult stage, genital development, including that of the testis, was normal, and no DSD was found in both groups (Fig. 2A-F). There were no significant differences in serum testosterone, semen quality (sperm density and proportion of immotile sperm), and seminiferous tubule morphology between the two groups (Fig. 2G-K). These results suggest that *Lhcgr*^W495X/+^ male offspring showed normal phenotype in the adult stage.

There was no significant difference in the mRNA expression of steroidogenic genes *(Lhcgr*, *Star*, *Cyp11a1*, *Hsd3b2*, *Cyp17a1*, and *Hsd17b3*) in adult offspring between the two groups (*n* = 5, *p* > 0.05) (Fig. 2L-Q). WB analysis also confirmed that there were no significant differences in the expression of LHCGR, HSD3B2, and HSD17B3 (*n* = 5, *p* > 0.05) (Fig. 2R-U). These results suggested that *Lhcgr*^W495X/+^ male offspring showed normal steroidogenic gene expression in the adult stage.

### Prenatal exposure to low-dose DEHP selectively induced DSD in Lhcgr^W495X/+^ male offspring by interfering with steroidogenic gene expression

#### Prenatal exposure to low-dose DEHP inhibited testosterone synthesis in Lhcgr^W495X/+^ neonatal male offspring by interfering with steroidogenic gene expression

**Fig. 3:**
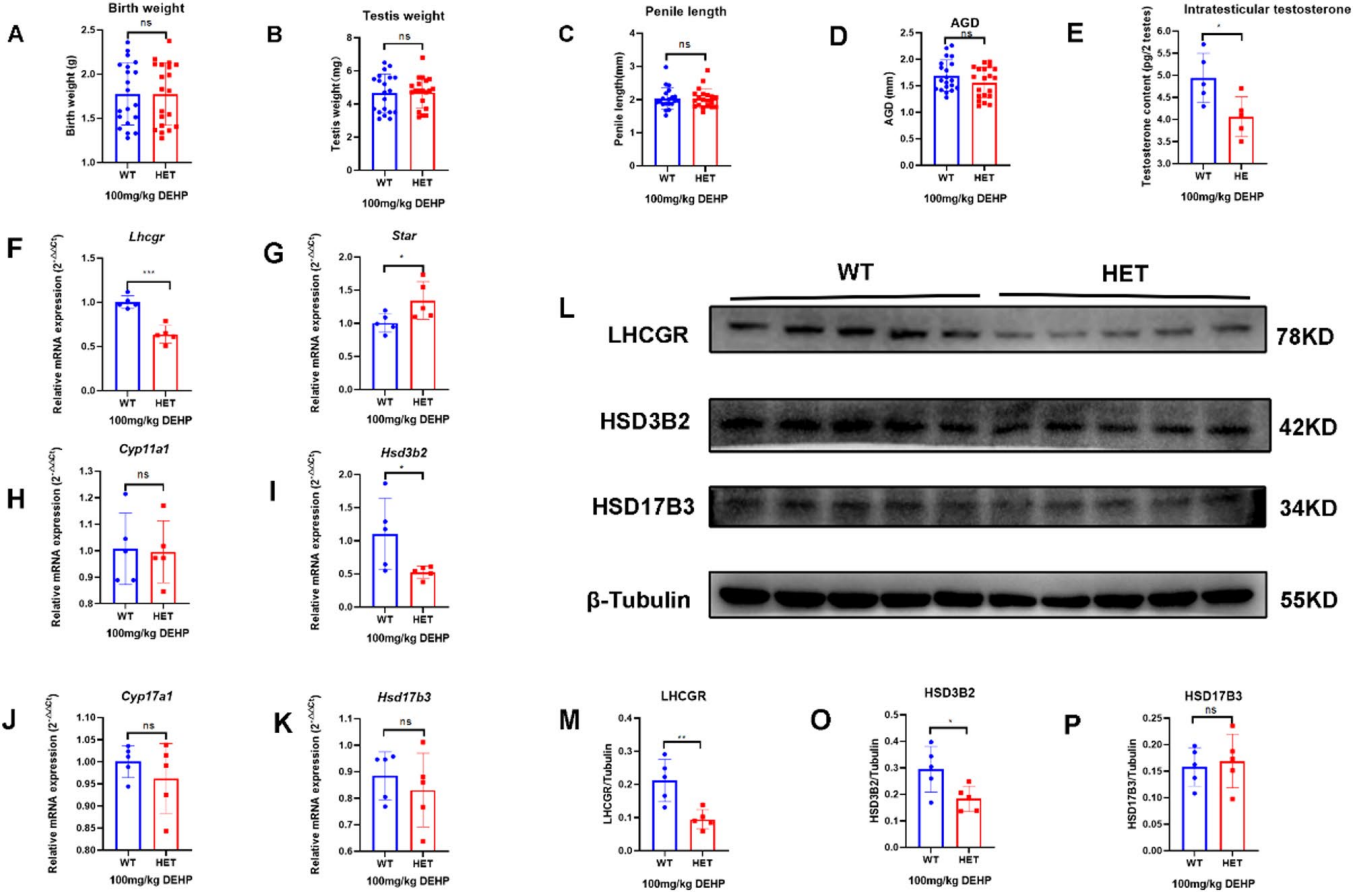
Prenatal exposure to low-dose DEHP inhibited testosterone synthesis in *Lhcgr*^W495X/+^ neonatal male mice by interfering with steroidogenic gene expression. **A**-**E**: the HET group showed reduced AGI and intratesticular testosterone. **F**-**K**: the mRNA expressions of *Lhcgr* and *Hsd3b2* in the HET group were lower than in the WT group. **L**-**P**: The expressions of LHCGR and HSD3B2 in the HET group were lower than in the WT group. ns: no significance; * *p* < 0.05; ** *p* < 0.01; *** *p* < 0.001

After prenatal exposure to low-dose (100 mg/kg/d) DEHP, no DSD was observed in the two groups in the neonatal stage. There were no significant differences in birth weight, testis weight, and penis length in neonatal male mice between the two groups. However, the AGD and intratesticular testosterone in the HET group were lower than those in the WT group (Fig. 3A-E). The results suggested that prenatal DEHP exposure affected genital development and testosterone synthesis in *Lhcgr*^W495X/+^ male offspring (Fig. 3A-E).

There was no significant difference in mRNA expression of *Star*, *Cyp11a1*, *Cyp17a1*, and *Hsd17b3* in neonatal offspring between the two groups (*n* = 5, *p* > 0.05). mRNA expression of *Lhcgr* and *Hsd3b2* in the HET group were lower than that in the WT group (*n* = 5, *p* < 0.05) (Fig. 3F-K). LHCGR and HSD3B2 expressions in the HET group were also lower than those in the WT group (*n* = 5, *p* < 0.05) (Fig. 3L-P). Because neonatal offspring were recently exposed to DEHP in utero, this experiment was similar to the cell experiment. These results indicated that prenatal DEHP exposure synergistically inhibited testosterone synthesis in *Lhcgr*^W495X/+^ neonatal male offspring by interfering with steroidogenic gene expression.

### Prenatal exposure to low-dose selectively DEHP selectively induced DSD and testicular dysfunction by interfering with steroidogenic gene expression in Lhcgr^W495X/+^ adult male offspring

**Fig. 4:**
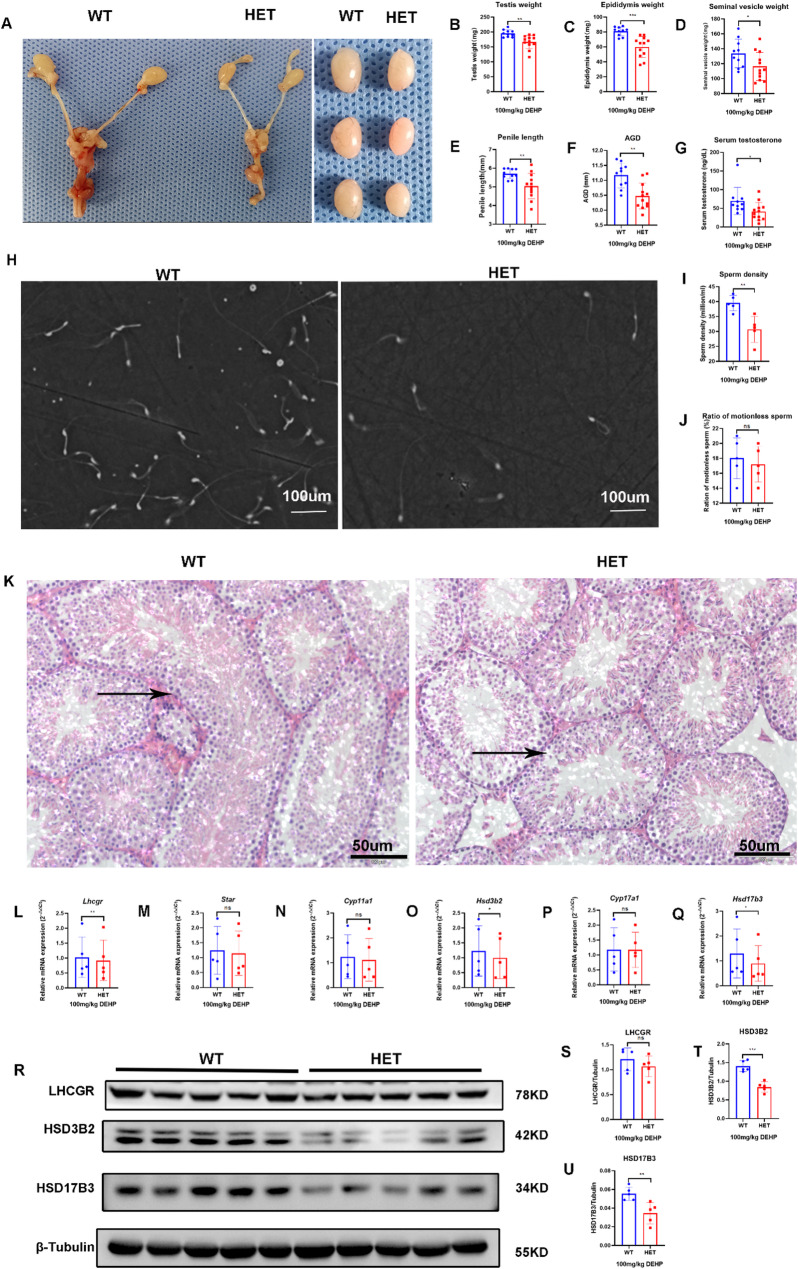
Prenatal exposure to low-dose DEHP induced DSD and testicular dysfunction by interfering with steroidogenic gene expression in *Lhcgr*^W495X/+^ adult male mice. **A**-**G**: *Lhcgr*^W495X/+^ adult male mice showed DSD, poorer genital development, and lower serum testosterone. **H**-**J**: *Lhcgr*^W495X/+^ adult male mice showed poorer semen quality (X100). K: HE staining (X200) of testicular tissue (→) showed poorer development of seminiferous tubules in the HET group. **L**-**Q**: The mRNA expressions of *Lhcgr*, *Hsd3b2*, and *Hsd17b3* in the HET group were lower than that in the WT group. **R**-**U**: LHCGR, HSD3B2, and HSD17B3 expressions in the HET group were lower than in the WT group. ns: no significance. * *p* < 0.05; ** *p* < 0.01; *** *p* < 0.001

This study found that the weight of the unilateral testis in WT mice without DEHP exposure was > 85 mg. If the testis was poorly developed, all the genital organs (epididymis, seminal vesicle, and prostate) were poorly developed. Therefore, if the weight of the unilateral testis was < 85 mg, it was categorized as DSD in this study. After prenatal exposure to low-dose DEHP, the situation changed. DSD was found only in the HET group in the adult stage. The incidence of DSD in the HET group (41.18%) was significantly higher than that in the WT group (0%, *p* = 0.04). Genital development, manifested as reduced testis weight, epididymis weight, seminal vesicle weight, penis length, and AGI, in the HET group was worse than that in the WT group (Fig. 4A-F). Serum testosterone (Fig. 4G) and semen quality (sperm density and proportion of immotile sperm, Fig. 4H-J) in the HET group were lower than those in the WT group. The seminiferous tubule in the HET group showed poorer development, which demonstrated as reduced tubular diameter and length, and dilatation of the lumen (Fig. 4K). These results suggest that prenatal exposure to low-dose DEHP synergistically induced DSD and testicular dysfunction (low testosterone and poor sperm quality) in *Lhcgr*^W495X/+^ adult male offspring. WT offspring showed nearly normal genital development and greater tolerance to prenatal DEHP exposure.

Although mRNA expression of *Star*, *Cyp11a1*, and *Cyp17a1* showed no difference, mRNA expression of *Lhcgr*, *Hsd3b2*, and *Hsd17b3* in the HET group were lower than that in the WT group (*n* = 5, *p* < 0.05) (Fig. 4L-Q). WB also confirmed that the expression of LHCGR, HSD3B2, and HSD17B3 was lower in the HET group than that in the WT group (*n* = 5, *p* < 0.05) (Fig. 4R-U). These results show the synergistic effect of prenatal DEHP exposure on steroidogenic gene expression in *Lhcgr*^W495X/+^ adult male offspring.

### Prenatal exposure to high-dose DEHP synergistically exacerbated DSD in Lhcgr^W495X/+^ male offspring by interfering with steroidogenic gene expression

#### Prenatal exposure to high-dose DEHP inhibited testosterone synthesis by interfering with steroidogenic gene expression in Lhcgr^W495X/+^ neonatal male offspring

**Fig. 5:**
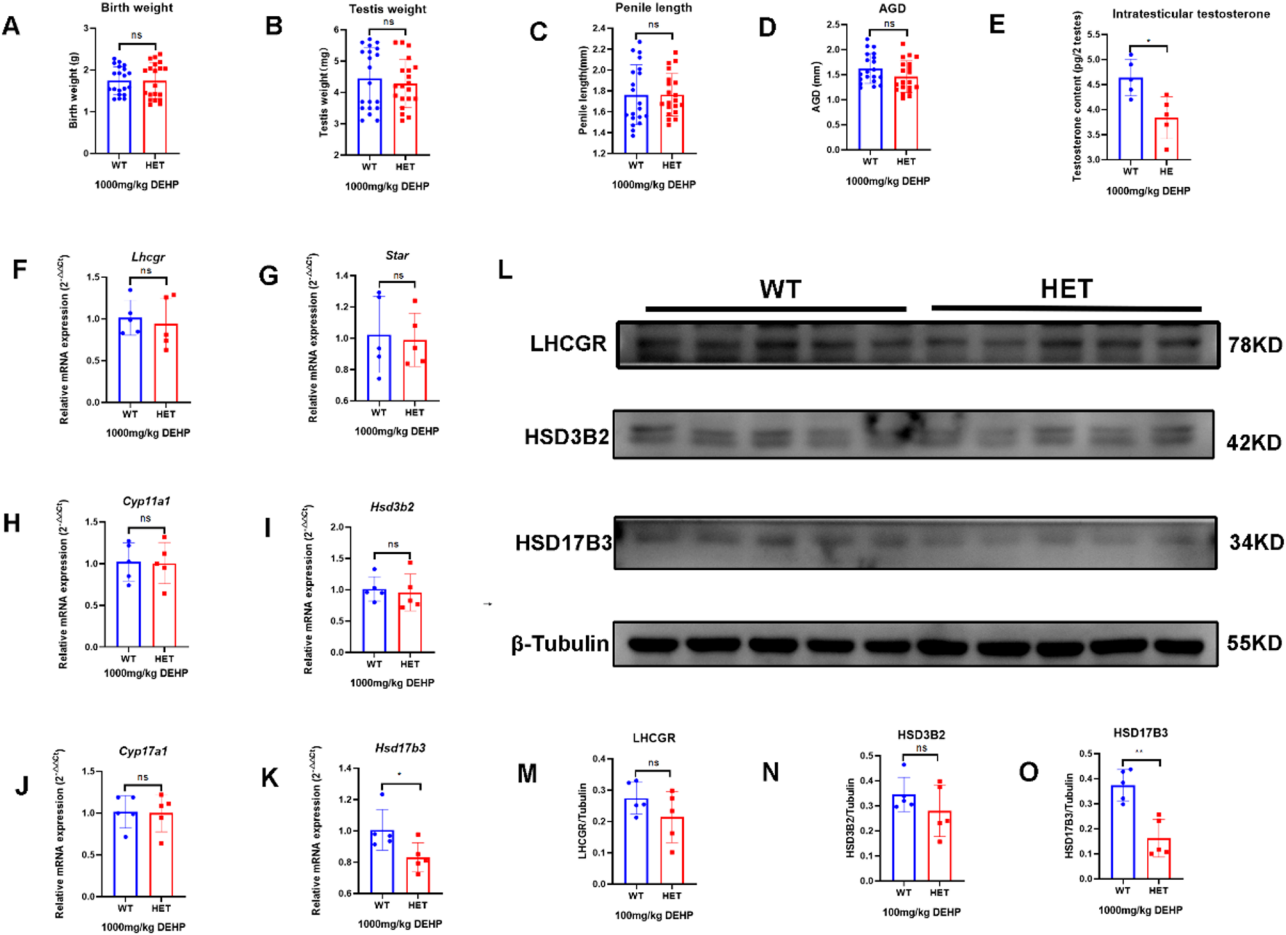
Prenatal exposure to high-dose DEHP inhibited testosterone synthesis by interfering with steroidogenic gene expression in *Lhcgr*^W495X/+^ neonatal male mice. **A**-**E**: the HET group showed reduced AGI and intratesticular testosterone. **F**-**K**: The mRNA expression of *Hsd17b3* in the HET group was lower than in the WT group. **L**-**O**: The expression of HSD17B3 in the HET group was lower than in the WT group. ns: no significance; * *p* < 0.05; ** *p* < 0.01; *** *p* < 0.001

After prenatal exposure to high-dose DEHP, there was no significant difference in birth weight, testis weight, and penis length between the two groups. However, the AGI and intratesticular testosterone in the HET group were lower than those in the WT group (Fig. 5A-E). Previous studies had verified that prenatal exposure to high-dose DEHP inhibited genital development in WT mice [29, 30]. Thus, our results indicated that prenatal exposure to high-dose DEHP affected genital development and testosterone synthesis in WT and HET male offspring. However, developmental stunting was relatively more severe in HET male offspring.

There was no difference in the mRNA expression of *Lhcgr*, *Star*, *Cyp11a1*, *Cyp17a1*, and *Hsd3b2* in neonatal male offspring between the two groups (*n* = 5, *p* > 0.05). However, the mRNA expression of *Hsd17b3* in the HET group was lower than that in the WT group (*n* = 5, *p* < 0.05) (Fig. 5F-K). The expression of HSD17B3 in neonatal male mice in the HET group was also lower than that in the WT group (*n* = 5, *p* < 0.05) (Fig. 5L-O). These results further verified prenatal DEHP exposure synergistically inhibits testosterone synthesis by interfering with steroidogenic gene expression in *Lhcgr*^W495X/+^ offspring.

#### Prenatal exposure to high-dose DEHP synergistically exacerbated DSD and testicular dysfunction in Lhcgr^W495X/+^ adult male offspring by interfering with steroidogenic gene expression

**Fig. 6:**
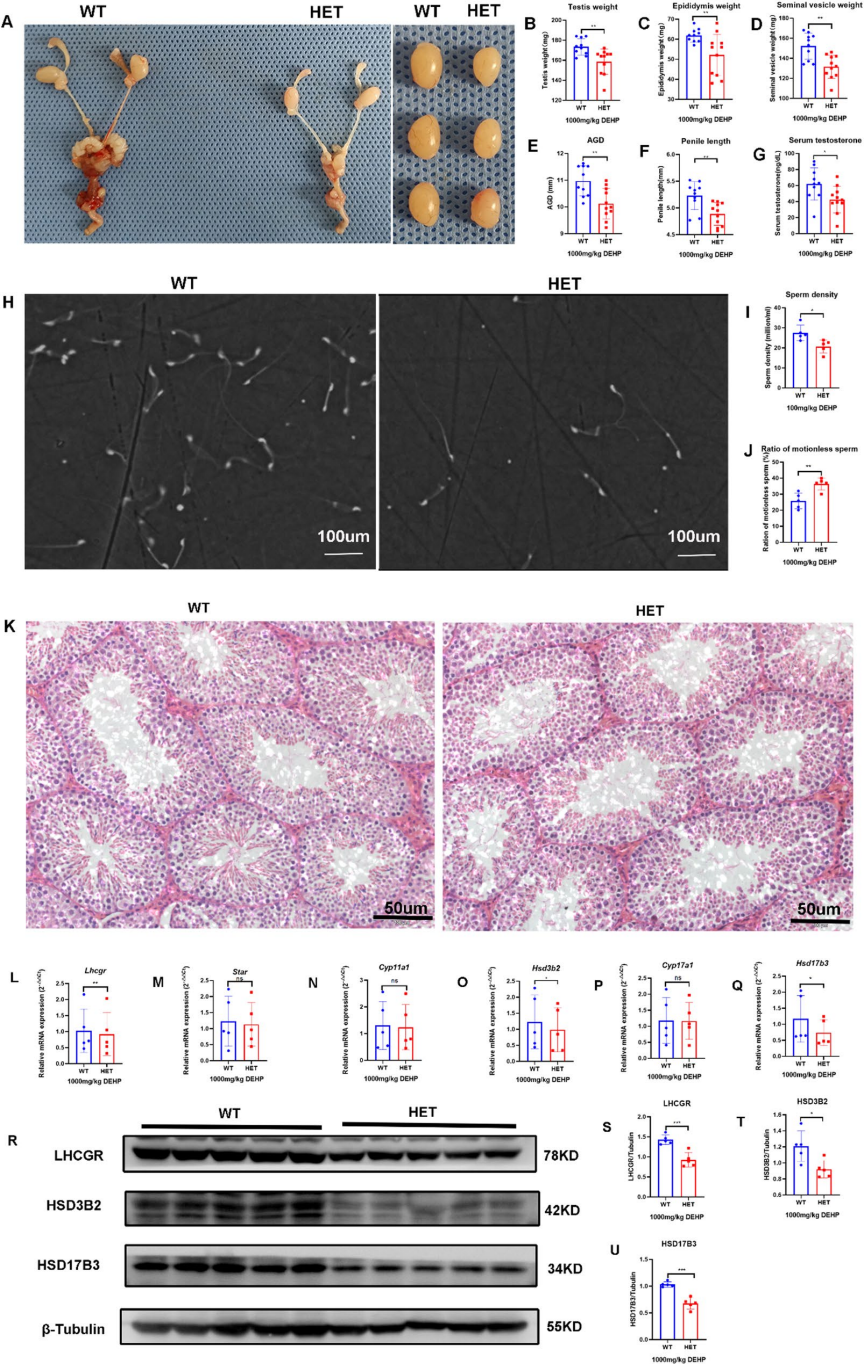
Prenatal exposure to high-dose DEHP synergistically aggravated DSD and testicular dysfunction in *Lhcgr*^W495X/+^ adult male mice by interfering with steroidogenic gene expression. **A**-**G**: the genital development and serum testosterone were worse in the HET group (X100). **H**-**J**: the semen quality was poorer in the HET group. **K**: HE staining (X200) of testicular tissue (→) showed that the development of seminiferous tubules was poorer in the HET group. **L**-**Q**: The mRNA expressions of *Lhcgr*, *Hsd3b2*, and *Hsd17b3* in the HET group were lower than that in the WT group. **R**-**U**: LHCGR, HSD3B2, and HSD17B3 expressions in the HET group were lower than in the WT group. ns: no significance; * *p* < 0.05; ** *p* < 0.01; *** *p* < 0.001

After prenatal exposure to high-dose DEHP, DSD was observed in both groups in adult offspring, but the incidence of DSD in the HET group (66.7%) was significantly higher than that in the WT group (16.7%, *p* = 0.03). Genital development in the HET group was also worse than that in the WT group, which manifested as reduced testis weight, epididymis weight, seminal vesicle weight, penis length, and AGI (Fig. 6A-F). Serum testosterone (Fig. 6G) and semen quality (Fig. 6H-J) were lower in the HET group than those in the WT group. The seminiferous tubule was shorter, and the internal diameter of the seminiferous tubule was wider in the HET group compared to those in the WT group (Fig. 6K). These results showed that prenatal exposure to high-dose DEHP induced DSD and testicular dysfunction in both groups. However, DSD was more severe in the HET group, thus further verifying that prenatal DEHP exposure synergistically aggravates DSD and testicular dysfunction in *Lhcgr*^W495X/+^ adult male offspring.

Although the mRNA expression of *Star*, *Cyp11a1*, and *Cyp17a1* showed no difference, the mRNA expression of *Lhcgr*, *Hsd3b2*, and *Hsd17b3* were lower in the HET group than that in the WT group (*n* = 5, *P* < 0.05) (Fig. 6L-Q). WB also confirmed that the expression of LHCGR, HSD3B2, and HSD17B3 in the HET group were lower than that in the WT group (*n* = 5, *p* < 0.05) (Fig. 6R-U). These results further verified that prenatal DEHP exposure synergistically induces DSD by interfering with steroidogenic gene expression in *Lhcgr*^W495X/+^ adult male offspring. It also suggested that the exposure dose impacted the synergistic effect on the interference with steroidogenic gene expression.

## Discussion

This study originally constructs a DSD model involving both genetic and environmental factors, *Lhcgr*^*W495X/+*^ male mice with prenatal DEHP exposure, to unravel the pathogenic modes of DSD. While genetic variants were detected abundantly in DSD, most of which are nonpathogenic [10, 31–33]. Epidemiological investigations have indicated that the exposure dose of DEHP in humans is significantly lower than that in animal models [34–37]. Previous studies, along with this one, confirm that prenatal exposure to low-dose DEHP does not induce DSD in WT male mice [29, 38, 39]. Consequently, the concept of DSD induced solely by environmental factors, whether of low or high dosage, or by nonpathogenic variants, does not align with clinical practice. The animal model integrating both genetic and environmental factors provides a more clinically relevant framework for studying DSD. Heterozygous point-mutation of *Lhcgr* is a typical nonpathogenic genetic factor of DSD, while DEHP is a ubiquitous environmental factor of DSD. Therefore, *Lhcgr*^W495X/+^ male mice exposed to prenatal DEHP represent an ideal model for studying gene-environment synergies, providing critical insights into the clinical relevance of DSD pathogenesis. A study demonstrated a synergistic induction of spermatogenesis disorders in male rats by a high-fat diet and low-dose DEHP [40]. Another study also revealed a significant increase in the incidence of testicular abnormalities resulting from the synergistic effect of genistein and DEHP [41]. These DSD models are emblematic of the interaction between environmental factors. However, a DSD model elucidating the synergistic interplay between genetic and environmental factors has been conspicuously absent. Consequently, our DSD model involving *Lhcgr*^W495X/+^ male mice exposed to prenatal DEHP successfully bridges this gap.

This study sufficiently substantiates the theory that genetic variants and environmental toxicants synergistically induce DSD through a three-phase experimental paradigm. Firstly, we confirmed that nonpathogenic variants or low-dose exposure could not induce DSD individually. This study confirmed that *Lhcgr*^*W495X/+*^ male offspring without prenatal DEHP exposure exhibited a normal phenotype, in concordance with Mendel’s law and clinical observations [42, 43], thereby verifying that nonpathogenic variants cannot induce DSD on their own. Meanwhile, this study found that prenatal exposure to low-dose DEHP failed to induce DSD in WT male offspring. Subsequently, we preliminarily verified that genes and the environment synergistically induce DSD through *Lhcgr*^*W495X/+*^male mice with prenatal exposure to low-dose DEHP. This study found that prenatal low-dose DEHP selectively induced DSD in *Lhcgr*^*W495X/+*^ male offspring. In contrast, WT offspring exhibited greater tolerance to low-dose DEHP exposure and had no DSD manifestation. Lastly, we reaffirmed the synergistic effect through *Lhcgr*^*W495X/+*^male mice with prenatal exposure to high-dose DEHP. As a result, prenatal high-dose DEHP exposure caused DSD in both WT and HET groups. Notably, the DSD observed in *Lhcgr*^*W495X/+*^ male mice was more severe than that in WT mice. Thus, the theory positing that gene and environment interact to induce DSD is successfully validated.

Our investigation reveals the pivotal role of nonpathogenic variants in DSD. Nonpathogenic variants have been conspicuously identified in DSD cohorts [10, 31–33], including variants of uncertain significance (VUSs), benign variants, and likely benign variants [44]. However, the clinical significance of nonpathogenic variants remains uncertain due to three causes. Firstly, these nonpathogenic variants are frequently heterozygous, and according to Mendel’s law, heterozygous variants do not elicit autosomal recessive disorders. Secondly, the pathogenicity of nonpathogenic variants is challenging to establish through clinical studies alone. Thirdly, DSD has not been caused solely by nonpathogenic variants in animal models. To date, no animal model has elucidated the role of nonpathogenic variants in DSD. Given the significantly high incidence, it is postulated that these genetic factors play a fundamental role in DSD. According to our findings, low-dose DEHP exposure successfully induced DSD in the *Lhcgr*^W495X/+^ male offspring, with no observed effects in WT counterparts. Hence, nonpathogenic variants such as *Lhcgr* heterozygous mutations serve as genetic susceptibility factors that augment sensitivity to environmental factors.

This study further explores the impact of exposure dosage on the synergistic effect by utilizing *Lhcgr*^*W495X/+*^ male offspring with prenatal exposure to varying dose of DEHP. Subsequent observations found that DSD in *Lhcgr*^*W495X/+*^ offspring with high-dose DEHP exposure was more severe than in *Lhcgr*^*W495X/+*^ offspring subjected to low-dose DEHP exposure, and *Lhcgr*^*W495X/+*^ offspring without exposure. These findings underscore the critical role of prenatal DEHP exposure (environment) in inducing DSD in *Lhcgr*^*W495X/+*^ (gene) male mice and the dependency of the synergistic effect on exposure dosage. Given that humans are perpetually exposed to various EEDs, each potentially producing synergistic effects [40, 41], it is plausible that the cumulative exposure dose of EEDs in humans exceeds our current estimations. Consequently, EEDs must be considered pivotal contributors to the pathogenesis of DSD.

Guided by our new pathogenic theory, we propose a pathogenic mode of DSD: pathogenic variants directly cause DSD, while nonpathogenic variants of DSD act as genetic susceptibility factors and cause DSD following prenatal exposure to EEDs. According to the pathogenic mode, we propose several key recommendations to prevent DSD. Firstly, heightened attention should be directed towards nonpathogenic variants in clinical practice. Secondly, every effort should be made to minimize prenatal exposure to EEDs. Thirdly, fetuses carrying DSD variants, whether pathogenic or nonpathogenic, should receive heightened vigilance in avoiding prenatal EED exposure. Lastly, prenatal screening for DSD variants and the prevention of EED exposure may represent innovative strategies for DSD prevention.

Our study focused on the synergistic mechanism of interference with steroidogenic gene expression. We posited that, in theory, genes exist in a double-dose state, with paired chromosomes and double-stranded DNA. Heterozygous variants typically affect only one DNA strand or one chromosome of the pair, allowing the gene to express and fulfill its physiological function. Our study initially confirmed that steroidogenic gene expression, in *Lhcgr*^*W495X/+*^ male mice, did not exhibit significant differences, suggesting that haploinsufficiency was not the primary pathogenic mechanism. Previous studies have reported varied effects of prenatal DEHP exposure on steroidogenic gene expression, with some genes being down-regulated while others are up-regulated [13, 14, 26, 45, 46]. This study also found prenatal exposure to low-dose DEHP didn’t interfere with the steroidogenic gene expression in WT mice. We infer that WT male mice possess a double dose of *Lhcgr*, allowing for compensatory expression of *Lhcgr*, which aids in withstanding environmental toxicants to some extent. Conversely, *Lhcgr*^*W495X/+*^ male mice possess only a single dose of *Lhcgr*, rendering their *Lhcgr* expression less resilient to environmental influences and hence rendering them more susceptible to DSD. As a result, in *Lhcgr*^*W495X/+*^ male mice subjected to prenatal DEHP exposure at both low and high doses, steroidogenic gene expression, particularly *Lhcgr*,* Hsd3b2*, and *Hsd17b3*, was significantly reduced at the levels of RNA and protein. Our study suggests the hypothesis that genetic variants and environmental toxicants synergistically interfere with steroidogenic gene expression.

Furthermore, our study delved into the synergistic impact of prenatal DEHP exposure on testicular function in *Lhcgr*^*W495X/+*^ male mice. Male sexual dysfunction and infertility are foremost among male disorders. Male disorders are challenging to rectify and often irreversible in adulthood [2, 47]. A primary underlying reason for this challenge is ignoring the association between prenatal exposure and adult male disorders. Our study illuminated that the genital development, serum testosterone levels, and semen quality in *Lhcgr*^*W495X/+*^ male mice deteriorated significantly following prenatal DEHP exposure. Moreover, the pathological examination revealed that the development of seminiferous tubules in *Lhcgr*^*W495X/+*^ male mice was markedly impaired in comparison to WT mice post prenatal DEHP exposure. This suboptimal genital development can impair the formation and transport of sperm. As testosterone plays a pivotal role in promoting the development of seminiferous tubules, sperm formation, and sperm maturation [48], the observed testosterone deficiency may contribute to the reduced sperm quality in *Lhcgr*^*W495X/+*^ male mice exposed to prenatal DEHP. These results confirm that genetic variants and environmental toxicants synergistically induce testicular dysfunction. Consequently, our study establishes the vital importance of prenatal prevention for male disorders, particularly in those with variants of DSD.

Nonetheless, our study has some limitations. Firstly, this lacked cell experiments and used neonatal testes, which had been recently exposed to DEHP, to simulate in vitro experiments and explore the synergistic mechanism. Additionally, our observations only encompassed the neonatal and adult stages, rendering the long-term synergistic effects inadequately explored. Furthermore, our study’s proposed mechanism primarily centered on the interference with steroidogenic gene expression; Other mechanisms such as cell senescence and oxidative stress were not explored. Nonetheless, our DSD model, featuring *Lhcgr*^*W495X/+*^ male mice subjected to prenatal DEHP exposure, effectively validated the theory positing the synergistic induction of DSD by genetic and environmental factors, closely mirroring clinical scenarios. With the increasingly prevalent use of Next Generation Sequencing, the early detection of DSD variants and subsequent tailored measures to mitigate exposure to specific EEDs present a feasible avenue for preventing DSD. In these respects, our study maintains its completeness and carries substantial clinical significance.

## Conclusions

This study verifies the hypothesis that genetic variants (*Lhcgr*^W495X/+^) and environmental toxicants (DEHP) synergistically induce DSD, thereby elucidating the pathogenesis of DSD. Interfering with steroidogenic gene expression and thus reducing testosterone synthesis may be an important mechanism. This finding highlights the clinical imperative to minimize prenatal exposure to endocrine disruptors, particularly in pregnancies with variants of DSD.

## Supplementary Information

Supplementary Material 1

Supplementary Material 2

## Data Availability

No datasets were generated or analysed during the current study.
