# Supplementary figures and images for: Genetic variants (LhcgrW495X/+) and environmental toxicants (DEHP) synergistically induce DSD by interfering with steroidogenic gene expression
Source: Biol Sex Differ. 2025 Sep 26;16:70. doi: 10.1186/s13293-025-00753-0 (PMC12465185; doi:10.1186/s13293-025-00753-0)

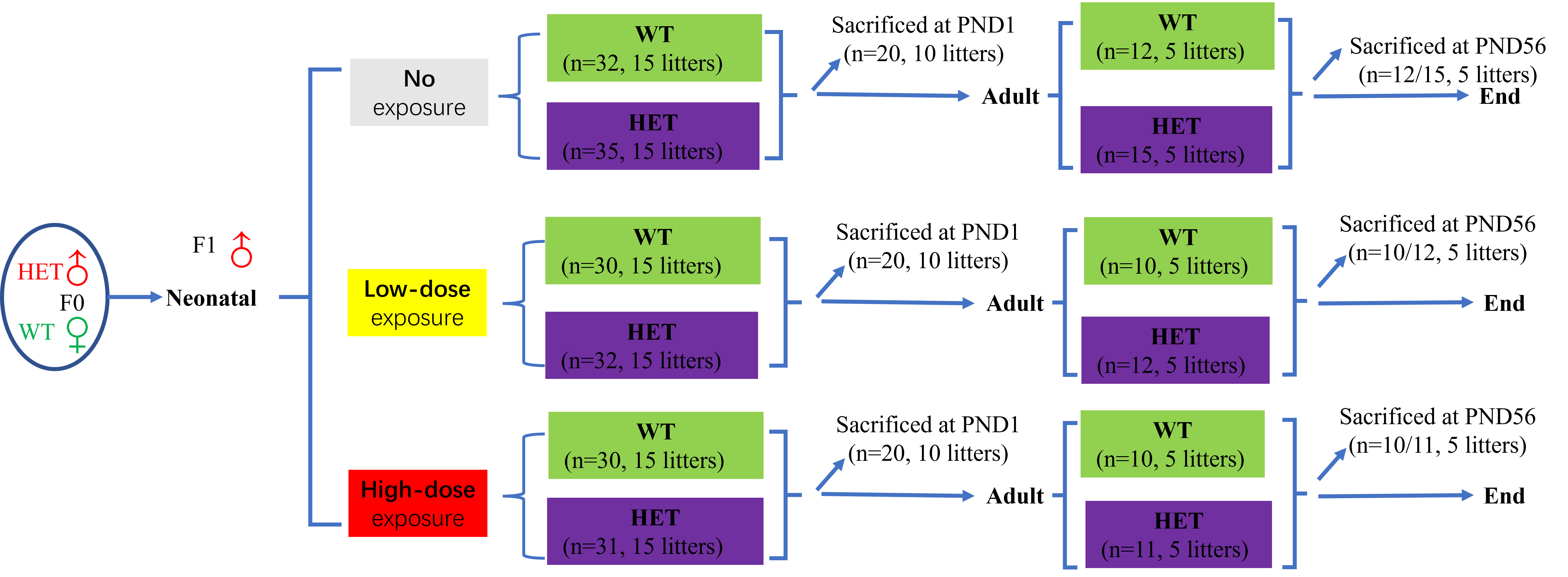

Supplement: Supplementary file 1 — Supplementary Material 1 [file 13293_2025_753_MOESM1_ESM.png]

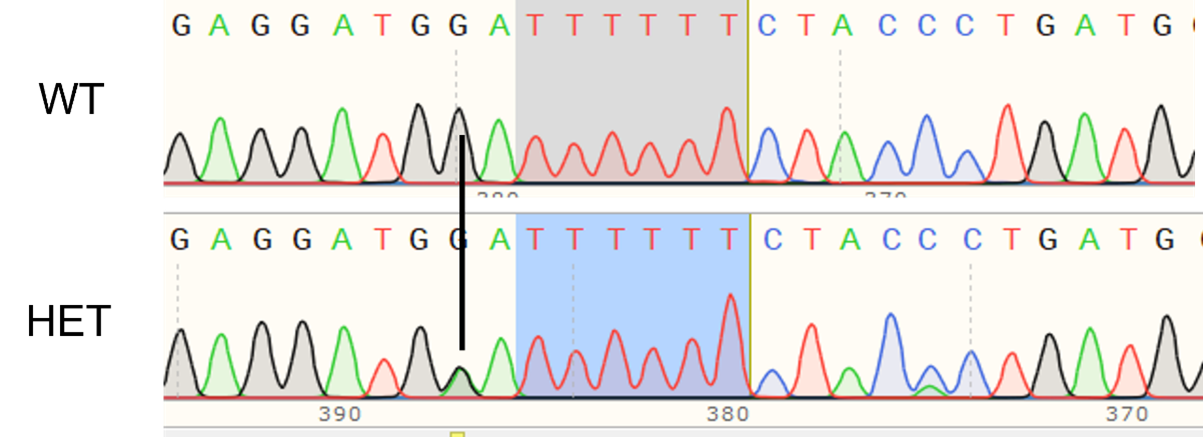

Supplement: Supplementary file 2 — Supplementary Material 2 [file 13293_2025_753_MOESM2_ESM.png]
